# Viral Coinfections Potentially Associated with Feline Chronic Gingivostomatitis in Cats with Feline Infectious Peritonitis

**DOI:** 10.3390/v17111505

**Published:** 2025-11-15

**Authors:** Jennifer Wenk, Marina L. Meli, Solène M. Meunier, Sandra Felten, Celia C. de Witt Curtius, Aline Crespo Bouzon, Ilaria Cerchiaro, Benita Pineroli, Anja Kipar, Stefan Unterer, Katharina Zwicklbauer, Katrin Hartmann, Regina Hofmann-Lehmann, Andrea M. Spiri

**Affiliations:** 1 Clinical Laboratory, Department of Clinical Diagnostics and Services, Center for Clinical Studies, University of Zurich, 8057 Zurich, Switzerland; jwenk@vetclinics.uzh.ch (J.W.); mmeli@vetclinics.uzh.ch (M.L.M.); celia.dewittcurtius@uzh.ch (C.C.d.W.C.); aline.crespobouzon@uzh.ch (A.C.B.); ilaria.cerchiaro@uzh.ch (I.C.); benita.pineroli@uzh.ch (B.P.); regina.hofmann-lehmann@uzh.ch (R.H.-L.); 2 Clinic for Small Animal Internal Medicine, Vetsuisse Faculty, University of Zurich, 8057 Zurich, Switzerland; smeunier@vetclinics.uzh.ch (S.M.M.); sandra.felten@uzh.ch (S.F.); stefan.unterer@uzh.ch (S.U.); 3 Institute of Veterinary Pathology, Vetsuisse Faculty, University of Zurich, 8057 Zurich, Switzerland; anja.kipar@uzh.ch; 4 LMU Small Animal Clinic, Centre for Clinical Veterinary Medicine, LMU Munich, D80539 Munich, Germany; k.zwicklbauer@lmu.de (K.Z.); hartmann@lmu.de (K.H.)

**Keywords:** FCoV, FIP, coinfection, FCGS, RT-qPCR, antiviral treatment, GS-441524, prognosis, immunodeficiency, FCV, retrovirus

## Abstract

Feline infectious peritonitis (FIP) is a fatal but now treatable disease in cats caused by feline coronavirus (FCoV). This study prospectively investigated viral coinfections in 100 cats diagnosed with FIP and subsequently treated with oral GS-441524 (Bova UK) and their influence on outcome, focusing on viruses potentially associated with feline chronic gingivostomatitis (FCGS). Cats were tested for feline leukemia virus (FeLV), feline immunodeficiency virus (FIV), feline calicivirus (FCV), feline herpesvirus (FHV), feline foamy virus (FFV), and feline gammaherpesvirus (FcaGHV1). Coinfections were identified at the following frequencies: FCV (27), FFV (22), FHV (6), FIV (4), FcaGHV1 (2), and FeLV (2, both progressive infections). FFV infection was significantly associated with FIV (p_F_ = 0.0021) and FHV (p_F_ = 0.0226) infection. FCGS was present in 25/97 cats with FCV infection being associated with FCGS (pF = 0.0032); no significant associa-tions were found for the other viruses and FCGS. The 42-day oral GS-441524 treatment’s success rate was 94% (five cats died, one relapsed). Coinfections did not significantly influence disease severity or treatment outcome, although the low number of cases for some pathogens warrants further investigation. However, advanced age was associated with treatment failure, potentially due to delayed diagnosis as FIP is considered to be less common in older individuals, or to age-related changes in immune function. In summary, viral coinfections, particularly with FCV, were common and should be considered in the clinical and hygienic management of cats with FIP.

## 1. Introduction

Feline infectious peritonitis (FIP) is a fatal disease affecting domestic cats, caused by feline coronavirus (FCoV) [1,2,3,4,5]. FIP can be associated with various clinical manifestations, including effusions, and neurological and ophthalmological signs. Studies have demonstrated that treatment with antiviral agents, such as GS-441524 or its prodrug (remdesivir), has made FIP a curable disease [6,7,8,9,10,11,12,13,14,15,16,17,18].

While effusion and fever are common findings in cats with FIP, additional clinical signs, such as conjunctivitis, ocular discharge, nasal discharge, and feline chronic gingivostomatitis (FCGS), have been occasionally reported [19,20,21]. However, some of these signs are likely attributable to coinfections rather than to FIP itself. With the advent of effective antiviral treatments and the possibility of curing FIP, the role of coinfections in clinical management and prognosis has gained attention. The present study focused on viral coinfections associated with FCGS, a common clinical problem in cats with FIP [22] that, to the authors’ knowledge, has so far not been scientifically addressed.

One of the viruses frequently associated with FCGS is feline calicivirus (FCV) [23]. Prevalence rates of FCV infection vary significantly, are lower in privately owned cats in small groups, and are higher in groups with three or more cats [24]. FCV infection can be asymptomatic or, typically, cause clinical signs including oral ulcerations, fever, and a reduced general condition in acute infection and FCGS in chronic infection. It can also manifest as a severe, virulent systemic disease with high lethality [23]. FCV was detected in up to 92% of oral mucosal samples from cats with FCGS [25,26]. However, experimental infection with FCGS-associated FCV isolates did not induce FCGS in the recipient cats, leading to the conclusion that FCGS is the consequence of an immune-mediated reaction to FCV [27]. Thus, despite its apparent association with FCGS, the precise role of FCV in the development of this condition remains inconclusive.

Feline foamy virus (FFV) and puma feline foamy virus (PFFV) are retroviruses and members of the *Spumaretrovirinae* subfamily, infecting felids worldwide [28,29,30]. FFV presumably establishes lifelong infections [31,32,33]. The clinical relevance of FFV infection in domestic cats remains unclear [31,34,35]. However, FFV has been suggested to exacerbate other retroviral infections and influence the progression of chronic infections or immunodeficiency syndromes [33,34,36]. Moreover, there has been evidence of an involvement in the genesis and clinical course of FCGS [28,29], and 60% of oral mucosal samples from cats with refractory FCGS tested PFFV-positive [25]. In vitro, PFFV hampered the production of new healthy gingival tissue [25]. In addition, FFV infection might facilitate the persistence of FCV in cats with FCGS [25]. The FFV antibody prevalence varies significantly in domestic cats, ranging from 30% to 80%, and it is influenced by factors such as age, sex, and geographical region [30,33,37,38,39,40,41], with 36% of Swiss cats being seropositive [40]. A PCR-based study to investigate the prevalence of FFV infections in Turkey found 20/200 cats (10%) to be positive [42].

Two other feline retroviruses, feline leukemia virus (FeLV) and feline immunodeficiency virus (FIV), can be associated with various clinical presentations, including immunodeficiency, depending on the disease course and stage [43]. In a large pan-European study, FeLV-infected cats were found to be affected more often by FCGS than uninfected cats [44]. FCGS is also a common finding in FIV-infected cats [45,46]. Progressive FeLV and FIV infections can lead to immunodeficiency, which increases the risk of secondary infections, FCGS, and tumor development, particularly lymphomas [43,47]. Both viruses could influence the severity of FIP manifestations and, thus, affect prognosis; their presence might also increase the overall risk of developing FIP [48,49,50]. To date, only one study has specifically examined clinical and laboratory parameters in cats strongly suspected of having FIP in conjunction with retrovirus infections. Among the 21 cats assessed, four were coinfected with FeLV and three with both FeLV and FIV, as determined by point-of-care testing. Mild anemia and lymphopenia were reported in these cats [50]; however, both of these conditions are also common in cats with FIP without coinfections [51]. The study mentioned neither if the cats with FIP and retrovirus infections received antiviral treatment nor the outcome [50].

Feline herpesvirus (FHV) and feline gammaherpesvirus 1 (FcaGHV1) both have the oronasopharynx as the site of shedding and might contribute to FCGS [52,53]. Both viruses could also be reactivated from their latent stage in cats that suffer from FIP. Approximately 80% of FHV-infected cats develop lifelong latent infections in neurons, with around 45% shedding the virus intermittently [54,55]. FHV can cause disease in young, elderly, or immunocompromised cats [52,56], and infection is particularly prevalent in shelters and breeding facilities [57,58]. In Switzerland, the prevalence of FHV infections detected by PCR was 9% in healthy cats [24]. FcaGHV1, being a gammaherpesvirus, can remain latent in B cells, especially in immunocompromised cats [59,60]. In a recent Swiss study, a substantial percentage of cats (10.5%) were found to carry FcaGHV1 [61]. The virus was more frequently detected in cats coinfected with FIV, emphasizing the interplay between these pathogens and their potential to contribute to the development and/or exacerbation of other diseases [61]. FcaGHV1 was also detected in other countries, indicating a widespread presence in the domestic cat population [62,63]. Recent studies in humans suggest that reactivation of the Epstein–Barr virus (EBV; a human gammaherpesvirus) during severe acute respiratory syndrome coronavirus 2 (SARS-CoV-2) infection could contribute to both the onset of autoimmunity and the prolonged immune-related symptoms seen in long COVID [64]. Furthermore, a link between EBV reactivation following SARS-CoV-2 infection and the onset of multisystemic inflammatory syndrome in children (MIS-C), a condition sharing similarities with FIP [65], has been reported [66].

The aims of this study were to determine the presence and importance of viral coinfections in cats diagnosed with FIP, with a focus on viruses potentially associated with FCGS. The infections were evaluated for their potential impact on disease severity at the time of FIP diagnosis as well as on the outcome of the subsequent antiviral treatment with oral GS-441524.

## 2. Materials and Methods

### 2.1. Animals

This study encompassed 100 client-owned cats diagnosed with FIP that subsequently underwent controlled antiviral treatment at the University Animal Hospital in Zurich, Switzerland, as part of a large, prospective bi-center study conducted in collaboration with the LMU Small Animal Clinic at LMU in Munich, Germany. The study was conducted in accordance with Swiss law and was approved by the veterinary office of the canton of Zurich (TVB ZH124/2022, issued 1 September 2022) and with institutional ethical approval (MeF-Ethik-2024-1 and -2024-14). Cat owners gave their written informed consent to participate. Cats were handled according to FearFree^®^ principles [67]. The IDs of the cats are listed in Table A1.

The cats were enrolled in the study on the day on which FIP was confirmed (day 1), before the start of treatment. FIP was diagnosed by board-certified specialists in accordance with the guidelines of the European Advisory Board on Cat Diseases (ABCD) [51]. This included the history, signalment, and clinical presentation of the cat, laboratory parameters, and at least one positive FCoV real-time reverse transcriptase quantitative polymerase chain reaction (RT-qPCR) result, in most cases with a high viral load in effusion, organ fine needle aspirates (FNAs), or cerebrospinal fluid (CSF), depending on the clinical FIP manifestation. Diagnosis was further supported by cytological findings and alpha-1-acid glycoprotein (AGP) values in the blood. Cats with all manifestations of FIP were included in this study. After FIP diagnosis, cats were enrolled in a prospective antiviral treatment study with oral GS-441524 (Bova AG, UK) at 15 mg/kg q24 h. The first 12 cats enrolled in the study (cats #001–#013) received treatment for 84 days, while the subsequent 88 cats (cats #015–#119) were treated for 42 days. Treatment duration was not assigned based on the specific FIP manifestations (i.e., neurological, ocular, and/or effusive). Treatment success was defined as clinical remission without relapse until day 183. The detailed results of the treatment study will be described elsewhere [68].

### 2.2. Signalment, History, and Assessment of Clinical Signs, Including FCGS

On day 1, a complete history was collated and a detailed clinical examination performed. For each cat, identification, signalment, place of origin, housing conditions (potential outdoor access, number of partner cats, and other animals in the household), history of previous diseases, and FeLV and FIV infection statuses were recorded. The Swiss Association for Small Animal Medicine vaccination guidelines and the ABCD vaccination recommendations were used to assess each cat’s vaccination status [69,70]. Data were collected from the cats’ vaccination records, and the owners had to fill out the history form. For the evaluation of FCGS, a physical examination of the oral cavity and photographic documentation were obtained to score the severity of FCGS using a published oral health score card: grade 1, moderate gingivitis only, no calculus; grade 2, moderate-to-marked gingivitis and calculus; grade 3, mild-to-moderate gingivitis with periodontitis; and grade 4, severe periodontitis [71]. Since most cats were awake during the oral examination, often only the rostral and alveolar/buccal regions could be fully evaluated. To assess the severity of the disease, the Karnofsky Score was used, comprising a clinician score (veterinarian-assessed) and a quality-of-life score (owner-assessed) [72] (Table A2). As many owners did not provide values for the quality-of-life score, this dataset was incomplete and only the clinician score (on a scale up to 50 points) was used for final evaluation.

### 2.3. Sample Collection and Processing

To assess coinfections, oropharyngeal cytobrush samples (OPCs) were collected using endocervical sampling brushes (Deltalabs S.L.U., Barcelona, Spain) rolled over the hard palate and the tongue of the cat. Samples were obtained from 97 cats; in 3 cats the collection was not possible. Brushes were transferred to sterile 1.5 mL microtubes (Sarstedt AG & Co. KG, Nümbrecht, Germany) with 300 μL of DNA/RNA shield (Zymo Research Europe GmbH, Freiburg i. B., Germany) and stored at −80 °C.

Blood samples were collected from all 100 cats, from the *vena jugularis*, *vena cephalica,* or *vena saphena medialis* by using stress-reduced procedures, including lidocaine ointment (Emla^®^ Crème 5% (Lidocainum 25 mg, Prilocainum 25 mg), Aspen Pharma Schweiz GmbH, Baar, Switzerland), in line with FearFree^®^ principles [67]. Ethylenediaminetetraacetic acid (EDTA)-anticoagulated blood was used for hematology and total nucleic acid (TNA) extraction. Native whole blood was centrifuged at 2000× *g* for 10 min to obtain the serum samples used for clinical chemistry, including acute-phase proteins, and for antibody/antigen testing.

### 2.4. Hematology, Serum Proteins, and Acute Phase Proteins

A complete blood cell count was performed using a Sysmex XN-1000 V (Sysmex, Kobe, Japan), validated for cats [73]. The analysis included red blood cell counts, reticulocyte counts, hemoglobin concentrations, red blood cell indices, platelet counts, and total white blood cell counts. Clinical chemistry profiles, including total protein, albumin, globulin, and albumin-to-globulin ratio were measured on a Cobas c 501 (Roche Diagnostics AG, Rotkreuz, Switzerland). Serum amyloid A (SAA) was analyzed using the latex agglutination turbidimetric immunoassay reaction (LZ Test SAA, Eiken Chemical Co., Ltd., Tokyo, Japan) on a Cobas c 501 clinical chemistry analyzer (Roche Diagnostics AG). AGP was measured in serum and effusion samples as described [74].

### 2.5. Total Nucleic Acid (TNA) Extraction from Blood, Effusions, and Oropharyngeal Cytobrush Samples

All laboratory work involving materials for molecular analysis was performed under laminar flow hoods in designated laboratories for each sample preparation step. Oropharyngeal cytobrush samples were processed prior to total nucleic acid (TNA) extraction as described [44]. TNA was extracted from 100 µL of EDTA-anticoagulated blood, 200 µL of effusion or 200 µL of OPC supernatant. The extraction was performed using either a MagNA Pure 96 instrument and the MagNA Pure 96 DNA and Viral NA Small Volume Kit, a MagNA Pure LC2 instrument, the MagNA Pure LC Total Nucleic Acid Kit (Roche Diagnostics AG), or the Maxwell^®^ RSC 48 (with the Maxwell RSC Pathogene Total Nucleic Acid Kit (Promega Corporation, Madison, WI, USA)), according to the manufacturers’ instructions. For each batch of extractions, a negative control consisting of PBS without Ca^2+^ and Mg^2+^ (Life Technologies Ltd., Thermofisher Scientific Inc., Waltham, MA, USA) was included to monitor for potential cross-contamination. TNA samples were stored at −80 °C until further use.

### 2.6. Molecular Detection of Feline Viruses (Viral RNA/DNA or Proviral DNA)

(RT-)qPCR for different pathogens was performed on various sample materials, as indicated in Table 1. For FeLV, viral RNA from blood and OPC as well as proviral DNA and p27 antigen in blood were tested on day 1, day 42, and day 84 to confirm the FeLV disease course (regressive or progressive) in positive cats.

Viral nucleic acids were amplified using 5 μL of TNA and 20 µL of PCR (qPCR) mastermix (qPCR MasterMix Plus Low ROX, Eurogentec, Seraing, Belgium) for viral DNA or RT-PCR (qPCR) mastermix (AgPath-ID™ One-Step RT-PCR Reagents, Thermofisher Scientific Inc.) for viral RNA on an ABI PRISM 7500 Fast Real-Time PCR system (Applied Biosystems, Foster City, CA, USA) using thermal conditions as previously described (for details, see Table 2). Proviral and viral DNA and RNA copy numbers in the individual samples were determined by co-amplifying 10-fold serial dilutions of a corresponding DNA or RNA standard template. For FCV, two assays (FCV S1 and FCV S2) were performed to increase sensitivity [75] (Table 2). FCV loads were calculated using FCV S1, except for the two samples (#037 and #069) where only FCV S2 was positive.

The molecular assays for the detection of all feline viruses but FFV have been described before (Table 2). For FFV, the assays were developed as part of this study (qPCR and RT-qPCR). The primers and probe were designed using the Geneious Prime^®^ 2020.2.5 (https://www.geneious.com, Biomatters Ltd., Auckland, New Zealand). The FFV assay also detects PFFV, a genetically close relative first identified in pumas [28,29]. For clarity, in this study both are referred to as FFV. Primers and probes were synthesized by a commercial supplier (Microsynth, Balgach, Switzerland). For FFV, the primers and probe were developed as follows: forward primer 5′-TAG ACC CCC TAG ACA ACA ACA GC-3′, reverse primer 5′-TCC TCC ACT TCC ACG AGG AG-3′, and probe 5′-6FAM-TGT TCC CGA ACA GAG AGA TCA RAG AGG ACC-BHQ-1-3′.

To determine the optimal primer and probe concentrations for the FFV assay, the following concentrations were tested: primers 300 and 900 nM, probes 100 and 250 nM; resulting in eight combinations which were tested with the 10^5^ copies/5 uL reaction of the corresponding synthetic standard (GeneArt^®^ Strings™, Thermofisher Scientific Inc). The optimal combination of primer and probe concentrations was selected based on the lowest cycle threshold (CT) value as the relative measure of the concentration of the target in the PCR reaction and the highest delta Rn (ratio of the fluorescence emission intensity of the reporter dye to the fluorescence emission intensity of the passive reference dye by subtracting the baseline) [84,85].

For the RT-qPCR assay (FFV RNA), the reaction consisted of 5 µL of TNA, 12.5 µL of RT-PCR buffer (AgPath-ID™ One-Step RT-PCR Reagents, Thermofisher Scientific Inc.), 1 µL of RT-PCR enzyme mix (AgPath-ID™ One-Step RT-PCR Reagents, Thermofisher Scientific Inc.), the primer and probe concentrations listed in Table 2, and molecular-grade water to reach a total volume of 25 µL. For the qPCR assay (FFV Provirus DNA), the following components were used: 5 µL of TNA, 12.5 µL of DNA PCR (qPCR) mastermix (Eurogentec), and 0.25 µL of Uracyl-N-Glycosylase (Eurogentec), along with the primer and probe concentrations specified in Table 2 and molecular-grade water to reach a total volume of 25 µL.

For the FHV, 10-fold serial dilutions of synthetic DNA fragments containing virus-specific sequences (GeneArt^®^ Strings™, Thermofisher Scientific Inc.) were utilized as DNA standards, as detailed in Table 2. For FeLV, FCV-S1, and S2, as well as FcaGHV1, standards were used as previously published (Table 2). The (RT)-qPCR positive controls (standards) and negative controls (molecular-grade water) were run in parallel for each assay and PCR.

### 2.7. Detection of FeLV p27 Antigen

FeLV provirus-positive cats were tested for free p27 antigen in the serum using a sandwich ELISA as described previously [86]. Any value higher than 4% of the positive control was classified as positive [87]. Cats that tested positive on day 1 were tested at later time points (days 42 and 84) to determine the course of FeLV infection (progressive versus regressive).

### 2.8. Detection of FIV Antibodies

All blood samples obtained at day 1 were tested for FIV antibodies by Western blot, following established protocols [88,89,90,91]. Serum was diluted in a 1:50 ratio and incubated with peroxidase-labeled goat anti-cat immunoglobulin G (H + L) (1:1000; Jackson Immunoresearch Laboratories, West Grove, PA, USA). Each blot included a positive (FIV-positive cat) and a negative (buffer) control. Samples were considered positive if two bands (p15 and p24) were present [90]. Samples with one band were considered inconclusive, and the cats were retested at a later time point (day 84 or 183).

### 2.9. Statistics

All data were compiled in Microsoft^®^ Excel^®^ 2016 for Microsoft 365, version 2013 (Microsoft, Redmond, WA, USA) and analyzed using GraphPad Prism 8 software (GraphPad Software LLC, La Jolla, CA, USA) and non-parametrical tests. Differences between groups were assessed using the Mann–Whitney U test (p_MWU_). Frequencies were compared using Fisher’s exact test (p_F_). The correlation was evaluated using the Spearman test (p_S_). A *p*-value ≤ 0.05 was considered significant. For sample prevalences, correlation coefficients, and relative risk, 95% confidence intervals (CIs) were calculated.

### 2.10. Post-Mortem Examinations

All animals who died or were euthanized due to very poor clinical conditions were subjected to a full post-mortem examination with subsequent histological examination.

## 3. Results

### 3.1. Characterization of the Study Population

**Figure 1:**
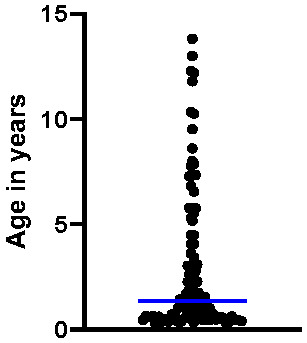
Age distribution of the 100 study cats. The blue line indicates the median age (1.4 years).

One hundred client-owned cats were included in this study. The cohort consisted of 13% female intact, 23% female spayed, 17% male intact, and 47% male neutered cats. The median age was 1.4 years, with a range from 0.3 to 13.8 years (Figure 1). A total of 61 cats were younger than 2 years, and 23 cats were older than 5 years.

**Figure 2:**
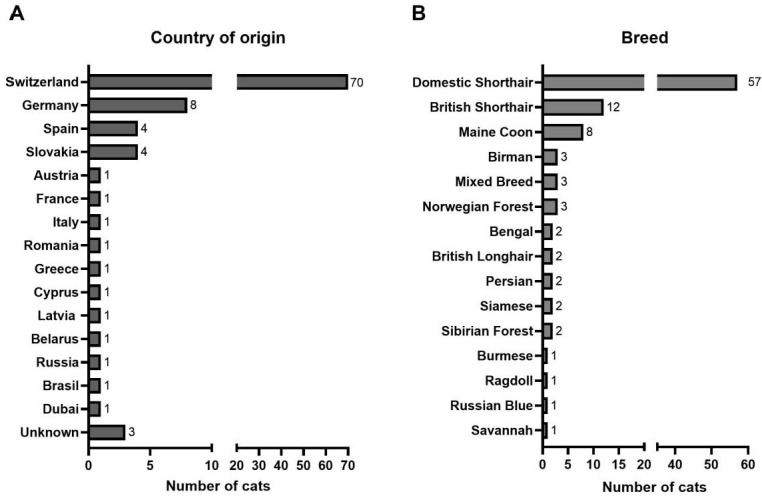
Country of origin (**A**) and breed (**B**) of the 100 study cats.

Switzerland was the country of origin of 70 of the cats, while 27 cats came originally from abroad; for three cats, the origin was unknown (Figure 2A). The study cohort included 57 domestic shorthair cats and 43 pedigree cats, with British Shorthair and Maine Coon being the most common breeds (Figure 2B).

The majority of the cats were up-to-date with their vaccinations for FCV and FHV (69/96, 72%) on the day of FIP diagnosis, according to the vaccination guidelines [69,70]. For five cats, the vaccination status was unknown (Table A1). Seven cats had been vaccinated against FCV and FHV within four weeks before sample collection at day 1.

In 91 of the 100 cats, effusions were diagnosed based on diagnostic imaging. Of the 91 cats, 76 had abdominal effusion, six presented with pleural effusion, and nine with bicavitary effusions. All cats with pleural and bicavitary effusions could be sampled, as well as 64/76 with abdominal effusions to confirm FIP diagnosis by FCoV RT-qPCR. In 12 cats with abdominal effusion, no sample was procurable. Instead, FNAs were collected from a lymph node, or a lymph node plus the liver and spleen, or a lymph node plus the kidney in 11 of these cats. From the remaining cat, CSF was collected for FIP diagnosis. Furthermore, from the nine cats without effusion, FNAs (*n* = 5; lymph node +/− liver and spleen) or CSF (*n* = 4) were collected for FIP diagnosis.

**Figure 3:**
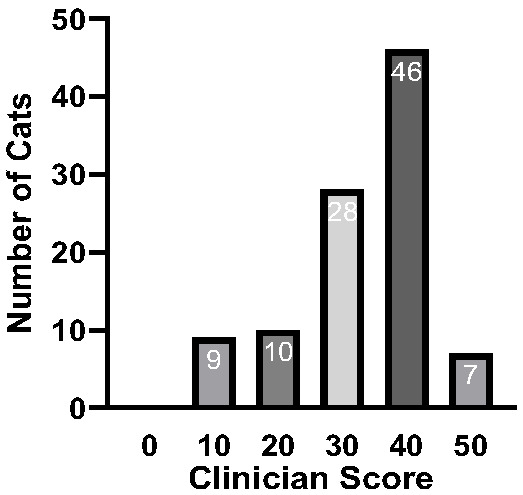
The severity of the disease is represented by the clinician score of the 100 study cats. Clinician score: 0 = dead; 10 = severely diseased; 20 = major changes in the general condition; 30 = medium changes in the general condition; 40 = minor changes in the general condition; and 50 = completely normal general condition.

The median clinician score (part of the Karnofsky’s score) for all 100 cats with FIP before the treatment’s start was 40, with scores ranging from 10 (severely diseased) to 50 (completely normal general condition) (Figure 3).

In 97 cats, the oral cavity could be examined, and three cats were not assessed. Seventy-two cats did not have FCGS and 25 were diagnosed with FCGS (26%), with a median FCGS grade of 1. Individual FCGS grades in the 25 cats with FCGS were as follows: 14 cats scored 1, three cats scored 2, five cats scored 3, and three cats scored 4. Cats with FCGS did not have fever (body temperature > 39 °C) significantly more often (19/25; 76%) than cats without FCGS (50/72; 69%; p_F_ = 0.6155). There was no significant association between the presence of FCGS and the age of the cats (p_Chi2_ = 0.4984). However, FCGS was more frequent in pedigree cats (15/41; 37%) than non-pedigree cats (10/56; 18%; p_F_ = 0.0590). A lower percentage of cats with FCGS was vaccinated with core vaccines (vaccinations up-to-date; 12/23; 52%) compared to cats without FCGS (58/71; 82%; p_F_ = 0.0113).

Overall, 34 cats had an albumin-to-globulin ratio < 0.4. The frequency of a decreased ratio did not differ significantly between cats with FCGS (9/25; 36%) and those without FCGS (24/71; 33%; p_F_ > 0.9999; for 3 cats, it was unknown whether they had FCGS; for one cat, no albumin-to-globulin ratio was available).

All cats underwent GS-441524 treatment, 12 cats for 84 days and 88 cats for 42 days. Eight cats either died or were euthanized prior to day 183, five of them due to their severe clinical condition, most likely associated with FIP (day of death: days 1–8), and three of them with other diseases according to necropsy (B-cell lymphoma, aspiration pneumonia, and *Toxoplasma gondii* encephalitis) (day of death: days 5–82). An age of above 2 years was associated with FIP-related death: all non-survivors were 4.5 years or older (#041, #049, #084, #091, and #106) whereas survivors were significantly younger (0/5 non-survivors < 2 years; 61/95 survivors < 2 years; p_F_ = 0.0076). An additional cat presented with a relapse 17 days post-treatment [92]. Overall, 94 cats achieved treatment success (94%). Detailed data regarding the treatment and its outcomes in the entire cohort will be published elsewhere [68].

### 3.2. Prevalence of Viral Coinfections

**Figure 4:**
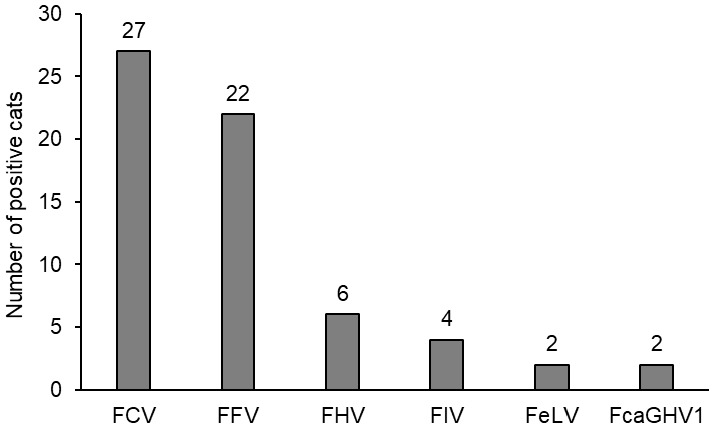
Number of cats with different viral coinfections. FCV, FFV, FHV, and FeLV were tested in oropharyngeal cytobrush samples (*n* = 97; from three cats (#001 to #003), no swabs were available). FIV, FeLV, and FcaGHV1 were tested from serum/blood (*n* = 100). FCV = feline calicivirus; FFV = feline foamy virus; FHV = feline herpesvirus; FIV = feline immunodeficiency virus; FeLV = feline leukemia virus; and FcaGHV1 = feline gammaherpesvirus.

**Figure 5:**
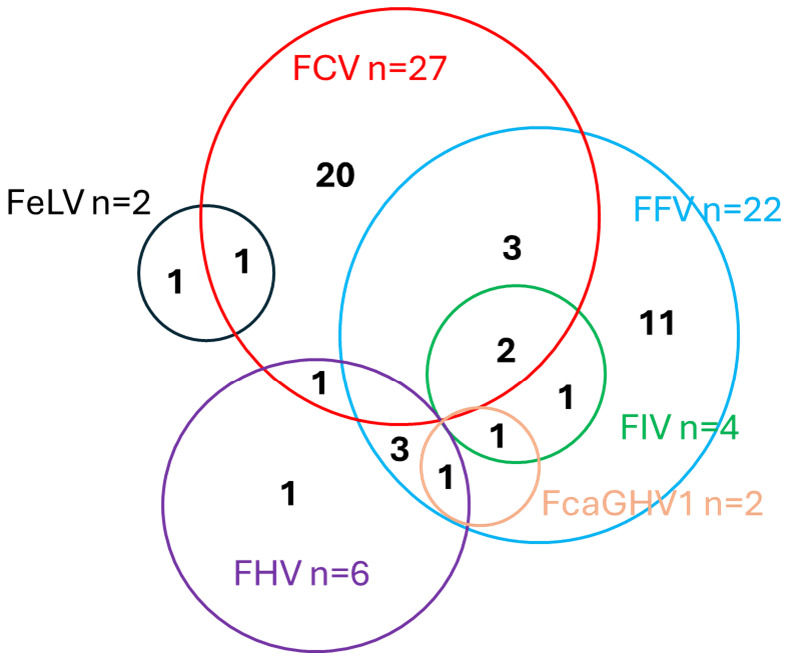
Venn diagram representing coinfections in the 100 cats with FIP. FCV = feline calicivirus; FFV = feline foamy virus; FIV = feline immunodeficiency virus; FeLV = feline leukemia virus; FHV = feline herpesvirus; and FcaGHV1 = feline gammaherpesvirus.

The 100 cats with FIP were evaluated for coinfections potentially associated with FCGS. Figure 4 provides an overview of the frequencies of all viral infections tested. The numbers of cats with several coinfections are presented in Figure 5.

FCV RNA was detected by RT-qPCR FCV in the OPC samples from 27 of the 97 tested cats (28%; Figure 4). For detailed results for all viruses and viral loads, refer to Table 3. In the 27 FCV-positive cats, the OPC samples from six cats tested positive for additional coinfections (Figure 5). Among the 27 FCV-positive cats, 17/27 (63%) had been appropriately vaccinated for their age, compared with 53/66 (80%) of the FCV-negative cats; this difference was not statistically significant. One of the FCV-positive cats had been FCV-vaccinated within 3 weeks before testing (#065) and two cats within 4 weeks (#008 and #042) (Table A1).

FFV infection was detected in 22 of the 97 tested OPC samples (23%; Figure 4; Table 3); 16 cats were positive for viral RNA and provirus, and six tested positive only for viral RNA. Viral RNA loads of positive cats were significantly correlated with provirus loads (p_S_ < 0.0001; r_S_ = 0.8517; 95% CI 0.6637–0.9385) but RNA loads were significantly higher than provirus loads (p_MWU_ < 0.0001) (Table 3). In the 22 FFV-positive cats, the OPC samples from 11 cats tested positive for additional coinfections (50%) (Figure 5).

Of the 97 OPC samples, six tested positive for FHV (Figure 4; Table 3). Of these, five tested positive for additional viruses (four were positive for FFV, one for FCV) (Figure 5).

Of the 100 tested cats, four were found to be FIV-positive as determined by Western blot (Figure 4; Table 3). The FIV-infected cats were 2.7, 3.0, 8.0, and 12.3 years old. All four cats had additional coinfections, i.e., the OPC samples tested positive for FCV and/or FFV (Figure 5). All FIV-positive cats also tested FFV-positive, and, thus, FFV-positive cats were significantly more frequently FIV-positive (4/22; 18%) compared to FFV-negative cats (0/75; 0%; p_F_ = 0.0021). One FIV-positive cat (#049), an 8-year-old male castrated domestic shorthair, died on day 8 of the treatment.

Two cats tested positive for FeLV proviral DNA, viral RNA (blood and OPC), and p27 antigen on day 1 (Figure 4; Table 3); these cats were subsequently followed through the treatment period and were found to be progressively infected as they tested provirus-, viral RNA-, and p27 antigen-positive also on days 42 and 84 after the start of antiviral treatment. The remaining 98 cats tested negative for FeLV provirus and viral RNA. None of the cats were regressively FeLV-infected. In one of the two progressively FeLV-infected cats, the OPC sample tested positive for FCV (Figure 5).

In two of the 100 tested cats, FcaGHV1 was detected in the blood (Figure 4; Table 3). Both cats had additional coinfections; both cats were FFV-positive, one was FHV- and the other one was FIV-positive as well (Figure 5).

### 3.3. Oropharyngeal Shedding of FCoV

OPC samples (*n* = 97) were tested for FCoV RNA as a means to assess viral oropharyngeal shedding. FCoV RNA was detected by RT-qPCR in the OPC samples from 35 cats (36%; 95% CI: 26.6–46.5%).

With FCV and FFV (each with >10 positive samples) as the coinfecting viruses with high prevalence, a potential association with oropharyngeal FCoV shedding was tested. No significant association was found (FCV: p_F_ = 0.6389; FFV: p_F_ = 0.0755).

Cats with FCGS did not have FCoV RT-qPCR-positive OPC samples significantly more often (10/24; 42%) than cats without FCGS (23/70; 33%; p_F_ = 0.4648; three cats were without OPC samples, and in three cats FCGS could not be assessed). Additionally, in cats with FCoV-positive OPC samples, FCoV loads did not differ significantly in cats with and without FCGS (p_MWU_ = 0.8982), and the FCGS scores were not associated with FCoV loads in FCoV-positive cats with FCGS.

### 3.4. Influence of Coinfections on Prevalence and Severity of FCGS

**Figure 6:**
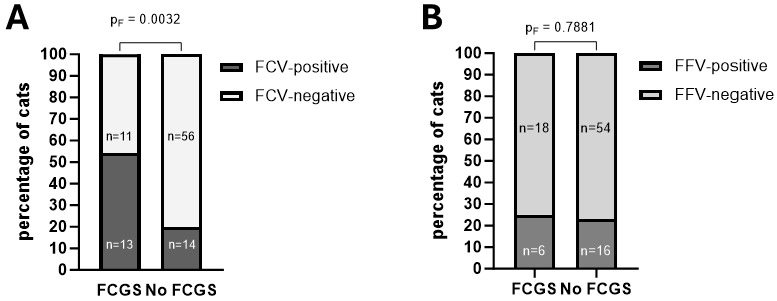
The occurrence of FCV infection (**A**) and FFV infection (**B**) was compared between cats with and without FCGS. Cats with FCGS were significantly more often FCV- but not FFV-positive than cats without FCGS (*p*-values are indicated in the figure). FCGS = feline chronic gingivostomatitis; FCV = feline calicivirus; and FFV = feline foamy virus.

OPC samples were available for 24 of the 25 cats diagnosed with FCGS (no sample from cat #002). Among these, 13 cats tested positive for FCV (54%), six for FFV (25%), and one each for FIV and FeLV (4.2%). In the group without FCGS, OPC samples were available for 70 of the 72 cats (no samples from cats #001 and #003). Of these, 14 tested positive for FCV (20%), 16 for FFV (23%), three for FIV (4.3%), and one for FeLV (4.3%). Thus, FCV was detected significantly more frequently in cats with FCGS (13/24; 54%) compared to those without (14/70; 20%; p_F_ = 0.0032; relative risk 2.9, 95% CI 1.5–5.6; Figure 6A), whereas the prevalence of FFV was similar between the 2 groups (Figure 6B). The other coinfections were not assessed due to the low number of positive cases.

Neither FCV nor FFV appeared to have an effect on FCGS severity in cats with FIP: among cats with FCGS, the grade did not differ significantly between FCV-positive (median grade 1) and FCV-negative cats (median grade 2; p_Chi2_ = 0.4468), nor between FFV-positive (median grade 2) and FFV-negative cats (median grade 1; p_Chi2_ = 0.2775).

Likewise, FCV-positive cats with FCGS did not show significantly higher FCV loads than FCV-positive cats without FCGS (p_MWU_ = 0.1014), and viral RNA loads did not differ between FFV-positive cats with and without FCGS (p_MWU_ = 0.2308).

Two cats with FCGS had coinfections with FCV and FFV (8.3%), one with FCV and FeLV (4.2%). Three cats without FCGS were coinfected with FCV and FFV (4.1%), and two of these were also FIV-positive (2.7%). No significant association was found between the presence of the FFV and FCV infections (p_F_ = 0.6014). Table 4 provides an overview of signalment, FCGS grades, and the prevalence of coinfections in each cat.

### 3.5. Disease Severity and Outcome

There was no significant difference in the clinician score between cats with and without FCV or FFV coinfections (FCV: p_Chi2_ = 0.2874; FFV p_Chi2_ = 0.4736). For FCV coinfection, the median clinician score was 30 and the score for cats without FCV infection was 40; for FFV, it was 40 regardless of coinfection.

Similarly, treatment success rates were comparable in cats with and without FCV or FFV coinfections (FCV: p_F_ > 0.9999; FFV: p_F_ = 0.6157). With FCV infection, 26/27 cats recovered (96%); without FCV infection, 65/70 cats recovered (93%). With FFV infection, 20/22 cats recovered (91%), without FFV infection, 71/75 cats recovered (95%).

FIV, FeLV, FcaGHV1, and FHV coinfections were not analyzed individually for their potential impact on clinician score or recovery rate because of the low number of positive cases; instead, they were combined and evaluated as a group. In total, there were four FIV-positive cats, two FeLV-positive cats, six FHV-positive cats, and two FcaGHV1-positive cats, including one cat each that was coinfected with FHV and FcaGHV1 and with FIV and FcaGHV1, respectively. Thus, the 12 cats positive for any of these viruses were compared with the 85 cats that tested negative for all four. Clinician scores did not differ significantly between cats with FIV, FeLV, FHV, or FcaGHV1 coinfections (median 40) and those without (median 40; p_Chi2_ = 0.8967). Recovery rates were also similar (92% vs. 94%; p_Chi2_ = 0.5574).

The GS-441524 treatment of the 46 cats coinfected with any of the viruses was successful in 44 (96%), and in 47 of the 51 cats (93%) without coinfection (p_F_ = 0.6801).

The potential influence of coinfections on AGP and SAA levels were evaluated to assess whether coinfections had an influence on the level of systemic inflammation. AGP levels were not significantly different in cats with any of the viral infections tested: FCV (median positive: 3400 µg/mL; median negative: 3553 µg/mL; p_MWU_ = 0.6854); FFV (median positive: 3290 µg/mL; median negative: 3606 µg/mL; p_MWU_ = 0.6873); and FIV/FeLV/FHV/FcaGHV1 combined (median positive = 3798 µg/mL; median negative = 3526 µg/mL; p_MWU_ = 0.4728).

Similarly, SAA levels were not significantly different in cats with any of the viral infections tested: FCV (median positive: 65.0 mg/L; median negative: 63.2 mg/L; p_MWU_ = 0.6964); FFV (median positive: 50.9 mg/L; median negative: 65.4 mg/L; p_MWU_ = 0.2676); and FIV/FeLV/FHV/FcaGHV1 combined (median positive = 62.4 mg/L; median negative = 65.0 mg/L; p_MWU_ = 0.9418).

AGP levels did not differ significantly between cats with any of the coinfections (*n* = 46, median 3392 µg/mL; one missing value, cat #027) and those without coinfections (*n* = 50, median 3554 µg/mL; p_MWU_ = 0.8409). Similarly, SAA levels were comparable between the two groups (median 64.8 mg/L vs. 64.3 mg/L; p_MWU_ = 0.6858).

## 4. Discussion

This study investigated viral coinfections in cats with FIP. With the advent of effective antiviral treatments, the potential impact of other common feline viruses on clinical presentation, disease severity, and treatment outcomes is gaining relevance. The present investigation focused on coinfections with six viruses potentially related to FCGS, which was quite commonly observed in cats with FIP alongside the typical clinical manifestations. Notably, this is the first report of FFV detection in Switzerland using molecular methods; until now only evidence of antibody presence was available [40].

The results show that viral coinfections are common in cats with FIP, with FCV (28%) and FFV (23%) being the most frequently detected viruses. However, coinfections appeared not to have an impact on disease severity and treatment outcome, as evaluated by the clinician score and the survival up to day 183, an important result that is relevant for the treatment of cats with FIP. Nevertheless, routine testing for common pathogens, such as FCV, remains advisable: it allows for the optimization of supportive care and, in the case of FCV, the implementation of appropriate hygienic measures to limit the virus’s spread [23]. For pathogens with low prevalence in this study, their potential impact on FIP disease progression might have been underestimated, and larger-scale studies including both FIP and non-FIP cats would be valuable to further clarify their role.

We also evaluated the FCV and FHV vaccination statuses of cats with FIP. So far, no data exists about core vaccination rates in cats with FIP. Most cats in the present study were up-to-date with their vaccinations according to established guidelines [69,70], with 72% appropriately vaccinated against FCV and FHV. This finding is consistent with a previous study in non-FIP cats that reported vaccination rates of 76% in cats suspected of FCV infection and 80% in healthy cats in Switzerland [24].

Approximately one in four cats with FIP (26%) showed signs of FCGS, although in most cases the severity was moderate (grade 1) [71]. Only a few cats showed marked FCGS. The latter was unrelated to FCoV shedding in saliva. To the authors’ knowledge, the prevalence of FCGS has not previously been specifically investigated in cats with FIP. A recent prospective UK cohort study reported an age-dependent prevalence of FCGS, with 25% of cats up to 12 months and 56% of cats aged 5–6 years being affected [71]. In the present study, there was no association between age and FCGS prevalence. However, pedigree cats more frequently presented with FCGS—certain purebred cats, such as Maine Coons, have been reported to be predisposed to early-onset or severe periodontal disease [93]. In fact, 6/8 Maine Coons in the present study presented with FCGS.

The pathogenesis of FCGS is incompletely understood, but chronic antigenic stimulation (e.g., persistent infections, hypersensitivity reactions, or allergies) may elicit dysregulated immune responses that predispose to disease [25]. Although vaccination provokes immune activation, several studies have reported a protective effect of vaccination on FCGS [94,95]. This is in line with our finding that cats with FCGS were less frequently vaccinated against core vaccine components (vaccination not up-to-date) than cats without FCGS. Hyperglobulinemia is a common feature of both FCGS and FIP and indicates immune activation [96,97,98,99]. The albumin/globulin ratio was also evaluated in the present study in cats presenting with FCGS. Only about a third of the cats had a decreased albumin/globulin ratio <0.4 typically found in cats with FIP [51], and no association was found between a decreased albumin/globulin ratio and the presence of FCGS. Larger studies incorporating the above-mentioned factors could help clarify a potential link between FIP and FCGS, a connection suggested by clinical observations in recent years [22] (and personal observation).

FCV is one of the viruses frequently linked to FCGS [25]. We detected FCV in 28% of cats with FIP, a prevalence higher than the 8% reported in healthy Swiss cats but lower than the 45% observed in cats clinically suspected of FCV infection, where FCGS was among the inclusion criteria [24]. Three FCV-positive cats (#008, #042, and #065) had been vaccinated 28 days prior to sampling, hence vaccine virus shedding cannot be excluded. Further viral sequencing would be warranted, but low viral loads may be limiting. Most FCV-shedding cats with FIP did not display clinical signs typical of acute FCV infection, such as oral or lingual ulcerations; however, 13 of the 27 FCV-positive cats presented with FCGS. Cats with FCGS were 2.9 times more frequently FCV-positive than those without, confirming earlier findings in Swiss cats where FCV-positive cats had an approximately four-fold higher risk of FCGS compared to FCV-negative cats [24]. In the present study, however, FCV infection did not appear to influence either the severity of FIP, the outcome of the treatment, or the severity of FCGS in affected cats. This may be due to the fact that FCGS is a multifactorial condition, involving various pathogens, host immune responses at both local and systemic levels, and environmental factors such as housing or stress [23,100].

FHV is not directly associated with FCGS; however, viral reactivation and oral shedding might occur in response to immunosuppression or stressful events [101,102,103,104], potentially contributing to FCGS development. FHV was detected in 6% of the cats with FIP. It needs to be noted that only OPC samples were available for FHV testing in the present study. While OPC samples are adequate for the detection of FHV, the sampling of additional sites, including the nose and conjunctiva, increases the detection rate [105,106]. This might partially explain why the prevalence in FIP cats was similar or even lower than the one reported previously for healthy cats in Switzerland (9%) using a combination of OPC, nasal, and conjunctival swabs [24]. Only one of the six FHV-infected cats exhibited moderate FCGS (grade 1). In accordance with this, the FHV loads in the six infected cats were low, indicating asymptomatic shedding or chronic infection, since only high viral DNA loads were shown to indicate active replication and involvement of the virus in acute clinical signs [81].

Feline retroviruses, particularly FeLV and FIV, have also been associated with FCGS, as their immunosuppressive effects promote opportunistic and secondary infections [107,108]. Indeed, progressive FeLV infection was found in two of the 100 cats with FIP (2%), and FIV infection was diagnosed in four (4%). These infection rates are in line with those reported in the Swiss cat population. The FeLV prevalence (antigen- or saliva viral RNA-positive) was at around 2–3% in both healthy and sick cats [44,109], while FIV was found in 4% of sick cats in an earlier study [110]. All four FIV-positive cats in the present study were older than 2 years (2.7, 3.0, 8.0, and 12.3 years), placing them outside the typical age range for cats with FIP [111,112]. This finding aligns with previous data from Switzerland showing that cats over 2 years of age are significantly more likely to be FIV-infected than younger cats [110]. FIV-associated immunosuppression might have contribute to an elevated replication of FCoV in the intestines, thus favoring the origination of FIP-associated mutations that may have subsequently led to the development of FIP in the older cats [48].

Only two cats suffered from progressive FeLV infection, and one of them showed grade 1 FCGS. Given these low numbers, no conclusions can be drawn regarding a potential influence of FeLV on the occurrence of FCGS in cats with FIP. Similarly, only one of the four cats with FIV infection showed FCGS (grade 3), and this cat was also coinfected with FFV and FcaGHV1.

Interestingly, regressive FeLV infection was not detected among the 100 cats with FIP in this study. This differs from earlier studies in Switzerland that reported a prevalence between 10% (2001) and 3% (2013–2016) for regressive FeLV infections [109] and allows speculation on the potential contribution of FIP to FeLV infection taking a progressive course, or on reactivation of regressive FeLV infections by the severe disease. From the patient histories, we could not gather information on the potential duration of FeLV infection prior to FIP development. However, since both cats were young (7 months and 16 months), primary progressive infection appears more probable. Interestingly, both cats were reported to be only living indoors, but one cat originated from Russia, where the infection risk might be increased due to higher prevalence rates [113]. The absence of cats with regressive FeLV infection in this study could also be due to the relatively small study population and the residential locations of the cats with FIP, which did not include areas with reportedly high FeLV prevalence [109]. Future studies investigating cats with concurrent FIP and FeLV infections are warranted to better understand the potential interactions between these viruses, particularly their roles in immune modulation through stimulation or suppression.

FFV was found in 23% of the cats. Some tested positive for viral RNA but not for proviral DNA, likely reflecting lower proviral loads compared with viral RNA loads, in some cases possibly approaching the lower limit of detection of the PCR. The 23% prevalence in the cats with FIP is similar to the 31% FFV prevalence observed in a separate cohort of 100 cats from stored biobank samples originally submitted for infectious disease diagnostics at the authors’ laboratory during the same period (personal communication A.M.S and R.H.-L.). Furthermore, antibody prevalence data reported approximately 20 years ago also align with these findings, with 36% of 99 cats admitted to a Swiss veterinary hospital testing FFV-positive [40]. A comparison of the molecular and antibody data appears valid for FFV, as the virus is believed to establish lifelong infection and since antibody responses persist for extended periods [38,114,115]. Some of the cats that tested molecularly positive for FFV were originally imported from abroad (Germany, Austria, Spain, Cyprus, Slovakia, and Belarus). It is unknown whether the cats already carried FFV in their country of origin or acquired it later, in Switzerland. Furthermore, while FFV infection in cats has been reported from Germany and Austria [30,116], the authors are not aware of investigations into FFV infection in the other countries of origin of these cats.

A potential association between FFV infection and FCGS was explored; however, the prevalence of FCGS was similar in FFV-positive (25%) and FFV-negative (23%) cats. There is evidence that FFV might hinder the healing processes of the oral mucosa [25], and that it might exacerbate other retroviral infections [33,34,36]. The present study revealed a significant association between FFV and FIV infection in cats with FIP. The association between the two viruses is more likely explained by shared transmission routes rather than by immune modulation or a synergistic effect of coinfection [36]. This hypothesis is also supported by the fact that young cats are less often infected than older cats, an observation that has been reported before for both FFV and FIV [33,38,117]. Interestingly, we also found an association between pedigree and FFV prevalence; indeed, pedigree cats were less frequently infected with FFV than non-pedigree cats.

We also found an association between FFV and FHV infections. This cannot readily be explained by similar transmission routes, since FFV is thought to be mainly transmitted via direct social contact (i.e., grooming) [38] while FHV can also be transmitted via droplets and indirectly through fomites [105]. To the authors’ knowledge, no studies have specifically investigated the coinfection, interaction, or epidemiological links between FFV and FHV in cats. However, one study suggested that other feline retroviruses, namely FIV and FeLV, might exacerbate the severity of oral disease when co-occurring with FCV or FHV [118].

Overall, we found no evidence that cats with FIP are more often coinfected with retroviruses. Additionally, they do not present more often with FCGS than cats without FIP. Interestingly, in the present study the therapy success rate was identical regardless of the retrovirus infection status (94%). Only one FIV and FFV coinfected cat died early, on day 8 of the therapy, due to its severe clinical condition, which according to the results of the post-mortem examination, was attributable to FIP alone. Thus, the current results suggest that, while the presence of a retrovirus infection (particularly FIV or FeLV) should be considered in the clinical management of cats with FIP, it should not be regarded as a reason to withhold treatment. This is also supported by a recent study that retrospectively evaluated the treatment outcome of FIP in FeLV-positive and FeLV-negative shelter cats [119]. The study found that FeLV-positive cats responded equally successfully to antiviral FIP therapy as FeLV-negative cats; however, their long-term survival was shorter.

Two of the one hundred tested cats with FIP were found to be FcaGHV1-infected (2%; 95% CI: 0.2–7.0%). This result is in line with a previous Swiss study using identical methods, which reported 6% (95% CI, 4.5–7.8%) in cats presented at veterinary clinics and 5.5% (95% CI, 1.8–12.4%) in stray cats [61]. The same study also reported a significant association of FcaGHV1 infection with FIV infection and a tendency for an association with FeLV infection [61]. While the two FcaGHV1-positive cats in the present study were not found to be FeLV-infected, one showed FIV coinfection. The small case number did not allow for statistical analyses. However, a higher likelihood of developing cancers, such as B-cell lymphoma, was reported for cats with FIV and FcaGHV1 coinfection [60]. The FIV- and FcaGHV1-coinfected cat is currently in good health and is being closely monitored during the long-term follow-up phase of the FIP treatment study. It might hence offer further information on the potential influence of FcaGHV1 in cats with FIP and vice versa.

Cats with FIP are typically young; according to the ABCD diagnostic tool, FIP is considered extremely likely in cats under two years of age and slightly less likely in cats older than five years [120]. In the present study, however, while the median age of affected cats was 1.4 years, 39% of cats were at least 2 years old, and 23% were 5 years or older. We highlight this finding to emphasize that clinicians should remain alert to FIP as a key differential diagnosis even in older cats when clinical history, presentation, and laboratory findings are suggestive of FIP. Interestingly, all 5cats that died due to severe disease in the early stages of treatment were over 4.5 years of age, the oldest being 10.3 years old. This observation needs to be validated by further studies but raises the question whether an older age is associated with a poorer outcome of FIP, whether it renders cats less susceptible to successful antiviral treatment, or whether age-related changes in immune function predispose for a more severe outcome of FIP.

## 5. Conclusions

This study provides detailed insight into viral coinfections potentially linked to FCGS in cats diagnosed with FIP. Coinfections were common, with FCV and FFV being most frequently detected, and many cats harboring multiple viruses. Encouragingly, there was no evidence that FIP disease severity or treatment success were affected by the observed coinfections. Nevertheless, testing for selected pathogens, such as FCV, FIV, and FeLV, remains essential for implementing hygienic measures and optimizing supportive care. The incidental finding that FIP is also a disease of older cats and that the advanced age of the affected cats is associated with therapy failure emphasizes that FIP should also remain a key differential diagnosis in older cats. The cats in this study are being prospectively monitored over two years to assess health status, blood parameters, and the potential long-term effects of coinfections on recovery, relapse risk, and susceptibility to immune-mediated or neoplastic diseases. Future investigations will also explore whether antiviral therapy with GS-441524 influences the course of concurrent infections.

## Figures and Tables

**Table 1:** (RT-)qPCR assays used in this study on the different sample materials obtained from 100 cats with FIP.

Virus	Target	Material	
		**OPC**	**Blood**
Feline coronavirus (FCoV)	Viral RNA	x	x
Feline calicivirus (FCV)	Viral RNA	x	
Feline foamy virus (FFV)	Viral RNA	x	
	Proviral DNA	x	
Feline leukemia virus (FeLV)	Viral RNA	x	x
	Proviral DNA		x
Feline herpesvirus (FHV)	Viral DNA	x	
Feline gammaherpesvirus (FcaGHV1)	Viral DNA		x

OPC = oropharyngeal cytobrush.

**Table 2:** (RT)-qPCR assays employed in this study.

Virus	Method	Target Gene	Amplicon Size (bp)	Forward Primer, Reverse Primer, Probe IDs	Concentration (nM)	Reference
FCoV	RT-qPCR	Orf 7b	103	FCoV1 (1147), FCoV2 (1072), FCoVp	900/300/300	[76,77]
FeLV	RT-qPCR, qPCR	FeLV LTR U3	131	exoFeLV-U3F2, exoFeLV-U3R2, exoFeLVU3p	900/300/200	[44,78]
FCV S1	RT-qPCR	Orf1 (polymerase)	95	FCV.forw, FCV.rev, FCV.p	300/900/250	[24,75,79]
FCV S2	RT-qPCR	Orf1 (polymerase)	120	FCV.1f, FCV.120r, FCV.26p	300/900/250	[75,80]
FHV	qPCR	Glyco- protein B	81	FHV.351f, FHV.431r, FHV.376p	400/400/80	[81,82]
FcaGHV1	qPCR	Glyco- protein B	113	FGHVF3, FGHVR3, FGHVP3	900/900/250	[61,83]
FFV	RT-qPCR, qPCR	gag	91	PFFV.2584f, PFFV.2674r, PFFV.2617p	300/900/250, 900/900/250	Newly established

Bp = basepairs; FCoV = feline coronavirus; FeLV = feline leukemia virus; FCV = feline calicivirus; FHV = feline herpesvirus 1; FcaGHV1 and FGHV = feline gammaherpesvirus; FFV = feline foamy virus; PFFV = puma feline foamy virus; Orf = open reading frame; LTR = long terminal repeat; exo = exogenous; U3 = unique region 3; and gag = group-specific antigen (gag polyprotein).

**Table 3:** Prevalence and loads of viral pathogens.

Virus	Prevalence (95% CI) [%]	Median Load OPC (Range) [Copies/OPC Sample]	Median Load Blood (Range) [Copies/mL Blood]
FCoV	36 (26.5–45.6)	5.4 × 10^2^ (1.7 × 10^1^–2.0 × 10^6^)	n.a.
FCV	28 (19.2–37.9)	2.1 × 10^5^ (1.1 × 10^0^–9.8 × 10^6^)	n.a.
FFV	23 (14.8–32.3)	Provirus OPC: 6.2 × 10^1^ (1.0 × 10^0^–6.1 × 10^3^) RNA OPC: 3.6 × 10^5^ (2.0 × 10^0^–1.5 × 10^7^)	n.a.
FHV	6 (2.3–13.0)	2.5 × 10^1^ (1.1 × 10^1^–1.4 × 10^4^)	n.a.
FIV	4 (1.1–9.9)	n.a	n.a
FeLV	2 (0.2–7.0)	RNA OPC: 6.8 × 10^7^ and 1.4 × 10^8^	Provirus blood: 3.6 × 10^8^ and 8.7 × 10^7^ RNA blood 1.4 × 10^9^ and 2.2 × 10^9^
FcaGHV1	2 (0.2–7.0)	n.a.	1.6 × 10^2^ and 1.2 × 10^3^

CI = confidence interval; OPC = oropharyngeal cytobrush sample; FCoV = feline coronavirus; FCV = feline calicivirus; FFV = feline foamy virus; FHV = feline herpesvirus; FIV = feline immunodeficiency virus; FeLV = feline leukemia virus; FcaGHV1 = feline gammaherpesvirus; and n.a. = not applicable.

**Table 4:** Heatmap of the different coinfections, oropharyngeal FCoV shedding, presence, and grade of feline chronic gingivostomatitis (FCGS), as well as the age, clinical score, and geographic origin of the cats.

Cat ID	Age (Years)	Clinician Score	FCGS (Grade)	FCoV Oral	FCV	FFV	FeLV	FIV	FHV	FcaGHV1	Number of Coinfections *	Origin of Cat
#001	1.8	20	0	n.s.	n.s.	n.s.			n.s.		(0)	CH
#002	1.7	40	3	n.s.	n.s.	n.s.			n.s.		(0)	ES
#003	3.4	30	0	n.s.	n.s.	n.s.			n.s.		(0)	CH
#004	0.8	40	1								2	BY
#006	5.2	30	1								1	DE
#007	1.3	10	4								1	CH
#008	0.8	40	0								1	AE
#009	1.1	40	0								0	DE
#010	4.1	50	2								0	CH
#011	0.6	40	0								0	SV
#012	0.7	40	0								1	CH
#013	0.9	40	0								0	CH
#015	1.1	50	1								2	CY
#017	10.3	10	2								1	CH
#018	1.8	50	1								1	ES
#022	0.3	40	0								0	CH
#023	7.8	40	0								0	CH
#024	1.3	40	1								0	ES
#025	1.4	40	1								2	RU
#027	1.1	30	0								0	CH
#028	0.7	50	0								1	CH
#029	1.2	30	0								0	SK
#031	5.8	30	4								0	CH
#033	0.5	40	0								1	CH
#034	7.3	40	0								1	CH
#035	7.3	20	2								2	AT
#036	2.6	20	0								1	CH
#037	0.6	10	0								2	CH
#038	7.3	40	1								0	CH
#039	12.3	40	3								3	CH
#040	0.4	30	0								0	CH
#041	4.5	10	n.k.								0	n.k.
#042	0.4	30	0								1	CH
#043	0.5	40	0								0	DE
#044	0.9	40	0								0	DE
#045	0.5	30	1								1	CH
#046	3.1	40	1								0	CH
#047	3.0	40	0								0	CH
#048	13.0	40	0								0	BR
#049	8.0	20	0								3	CH
#050	1.2	30	0								0	FR
#051	4.1	40	0								3	CH
#052	0.4	40	0								0	CH
#053	0.4	40	0								1	CH
#054	1.3	40	0								2	ES
#055	2.5	10	n.k.								0	CH
#056	1.1	40	0								1	CH
#057	3.3	40	0								0	IT
#059	2.8	40	0								0	CH
#060	0.5	40	0								0	CH
#061	1.4	40	0								0	CH
#062	0.6	20	3								1	CH
#064	1.4	10	0								0	CH
#065	0.4	30	0								1	CH
#066	2.3	30	0								1	CH
#067	7.2	40	0								1	CH
#069	0.5	20	0								1	GR
#070	5.6	30	0								0	CH
#071	1.9	30	0								0	LV
#073	0.6	50	1								1	DE
#074	0.5	30	0								1	DE
#075	6.6	40	3								0	CH
#076	0.6	30	0								0	CH
#077	0.6	30	0								0	CH
#078	3.6	30	0								1	CH
#079	0.6	30	0								0	CH
#080	1.5	40	3								0	CH
#081	1.0	10	4								1	CH
#082	8.6	20	0								1	CH
#083	0.4	20	0								1	CH
#084	10.3	10	0								0	CH
#085	0.7	40	0								0	CH
#086	2.7	30	0								3	SK
#087	0.5	30	0								0	CH
#090	1.0	40	0								0	CH
#091	5.7	10	n.k.								0	CH
#093	12.2	20	0								0	n.k.
#094	1.6	40	0								0	CH
#095	2.7	40	0								0	CH
#096	0.7	30	1								1	n.k.
#097	3.0	40	0								2	CH
#098	0.5	30	1								1	CH
#099	5.3	50	0								0	CH
#100	0.3	30	0								0	CH
#101	11.8	40	0								2	CH
#103	0.7	40	0								1	CH
#104	0.4	40	0								0	CH
#106	6.8	30	0								1	CH
#107	1.7	30	0								0	CH
#108	13.8	40	0								1	CH
#109	0.5	20	0								0	CH
#110	0.5	30	0								2	CH
#111	0.9	40	0								1	CH
#112	0.6	40	1								1	CH
#113	9.5	30	0								0	RO
#114	2.3	40	0								0	SK
#115	0.5	40	0								0	DE
#117	0.6	30	0								0	DE
#118	5.8	50	0								0	CH
#119	0.5	40	1								1	CH
		<2 years old							FCGS grade 0 (absent)			
		≥2 years old							FCGS grade 1			
		Clinician Score 10							FCGS grade 2			
		Clinician Score 20							FCGS grade 3			
		Clinician Score 30							FCGS grade 4			
		Clinician Score 40							No coinfection			
		Clinician Score 50							1 coinfection			
		(RT)-qPCR positive							2 coinfections			
		(RT)-qPCR negative							3 coinfections			

* The total number of coinfections are in parentheses, if not tested for all six infections. FCoV = feline coronavirus; FCV = feline calicivirus; FFV = feline foamy virus; FHV = feline herpesvirus; FIV = feline immunodeficiency virus; FeLV = feline leukemia virus; and FcaGHV1 = feline gammaherpesvirus. Origin: CH = cats born and raised in Switzerland; other countries—country of import: AT = Austria; BR = Brazil; BY = Belarus; CY = Cyprus; DE = Germany; ES = Spain; FR = France; GR = Greece; IT = Italy; LV = Latvia; RO = Romania; RU = Russia; SK = Slovakia; SV = Slovenia; n.k. = not known; and n.s. = no sample for molecular analyses.

## Data Availability

The authors confirm that the datasets analyzed during the study are available from the corresponding author upon reasonable request.

## Appendix A

**Table A1:** Age, vaccination status, and FCV/FHV (RT-) qPCR results in all the cats included in this study.

Cat ID	Age (Years)	FCV/FHV Vaccination Up-to-Date ^1^	Days Since Last Vaccination	FCV RT-qPCR	FHV qPCR
#001	1.8	Yes	167	Unavailable ^2^	Unavailable ^2^
#002	1.7	Unknown	Unknown	Unavailable ^2^	Unavailable ^2^
#003	3.4	No	1049	Unavailable ^2^	Unavailable ^2^
#004	0.8	No	66	**Positive**	Negative
#006	5.2	No	295	**Positive**	Negative
#007	1.3	No	406	**Positive**	Negative
#008	0.8	Yes	23	**Positive**	Negative
#009	1.1	Yes	246	Negative	Negative
#010	4.1	Yes	111	Negative	Negative
#011	0.6	Yes	434	Negative	Negative
#012	0.7	No	-	Negative	Negative
#013	0.9	Yes	110	Negative	Negative
#015	1.1	Yes	129	Negative	**Positive**
#017	10.3	No	1495	Negative	Negative
#018	1.8	Unknown	Unknown	Negative	Negative
#022	0.3	Yes	**18**	Negative	Negative
#023	7.8	Yes	155	Negative	Negative
#024	1.3	Yes	**20**	Negative	Negative
#025	1.4	No	123	**Positive**	Negative
#027	1.1	Yes	290	Negative	Negative
#028	0.7	Yes	106	**Positive**	Negative
#029	1.2	Yes	30	Negative	Negative
#031	5.8	Yes	344	Negative	Negative
#033	0.5	Yes	58	**Positive**	Negative
#034	7.3	No	218	Negative	**Positive**
#035	7.3	Ja	834	**Positive**	Negative
#036	2.6	Yes	343	Negative	Negative
#037	0.6	Yes	102	**Positive**	**Positive**
#038	7.3	Yes	505	Negative	Negative
#039	12.3	No	3271	Negative	Negative
#040	0.4	Yes	48	Negative	Negative
#041	4.5	Unknown	Unknown	Negative	Negative
#042	0.4	Yes	**22**	**Positive**	Negative
#043	0.5	Yes	63	Negative	Negative
#044	0.4	Yes	210	Negative	Negative
#045	0.5	Yes	70	**Positive**	Negative
#046	3.1	Yes	285	Negative	Negative
#047	3.0	Yes	269	Negative	Negative
#048	13.0	No	3018	Negative	Negative
#049	8.0	No	Unknown	**Positive**	Negative
#050	1.2	Yes	318	Negative	Negative
#051	4.1	Yes	118	Negative	**Positive**
#052	0.4	Yes	46	Negative	Negative
#053	0.4	Yes	68	**Positive**	Negative
#054	1.3	Yes	257	**Positive**	Negative
#055	2.5	Yes	93	Negative	Negative
#056	1.1	Yes	Unknown	Negative	Negative
#057	3.3	Yes	**10**	Negative	Negative
#059	2.8	Yes	**25**	Negative	Negative
#060	0.5	Yes	64	Negative	Negative
#061	1.4	Yes	370	Negative	Negative
#062	0.6	No	107	**Positive**	Negative
#064	1.4	No	323	Negative	Negative
#065	0.4	Yes	**14**	**Positive**	Negative
#066	2.3	Yes	186	**Positive**	Negative
#067	7.2	Yes	946	Negative	Negative
#069	0.5	Yes	30	**Positive**	Negative
#070	5.6	Yes	76	Negative	Negative
#071	1.9	Yes	81	Negative	Negative
#073	0.6	Yes	50	**Positive**	Negative
#074	0.5	No	65	Negative	Negative
#075	6.6	No	2311	Negative	Negative
#076	0.6	No	55	Negative	Negative
#077	0.6	Yes	83	Negative	Negative
#078	3.6	Yes	94	Negative	Negative
#079	0.6	Yes	51	Negative	Negative
#080	1.5	Yes	321	Negative	Negative
#081	1.0	Yes	259	**Positive**	Negative
#082	8.6	Yes	410	Negative	Negative
#083	0.4	No	30	**Positive**	Negative
#084	10.3	Unknown	Unknown	Negative	Negative
#085	0.7	Yes	164	Negative	Negative
#086	2.7	Yes	125	**Positive**	Negative
#087	0.5	No	68	Negative	Negative
#090	1.0	Yes	191	Negative	Negative
#091	5.7	Unknown	Unknown	Negative	Negative
#093	12.2	Yes	53	Negative	Negative
#094	1.6	Yes	138	Negative	Negative
#095	2.7	Yes	532	Negative	Negative
#096	0.7	No	-	**Positive**	Negative
#097	3.0	No	112	Negative	Negative
#098	0.5	No	69	**Positive**	Negative
#099	5.3	Yes	251	Negative	Negative
#100	0.3	No	380	Negative	Negative
#101	11.8	Yes	316	Negative	**Positive**
#103	0.7	Yes	153	Negative	Negative
#104	0.4	Yes	35	Negative	Negative
#106	6.8	Yes	68	Negative	Negative
#107	1.7	No	555	Negative	Negative
#108	13.8	Yes	214	Negative	Negative
#109	0.5	Yes	**16**	negative	Negative
#110	0.5	Yes	488	Negative	**Positive**
#111	0.9	Yes	133	**Positive**	Negative
#112	0.6	Yes	95	**Positive**	Negative
#113	9.5	Yes	492	Negative	Negative
#114	2.3	Yes	60	Negative	Negative
#115	0.5	Yes	75	Negative	Negative
#117	0.6	Yes	102	Negative	Negative
#118	5.8	Yes	275	Negative	Negative
#119	0.5	No	76	**Positive**	Negative

^1^ Protection against FCV and FHV requires two vaccinations 3–4 weeks apart, also in unprotected adults; the last kitten vaccination should be given at 16 weeks and a final vaccination at 10 to 16 months; in adults, booster vaccinations should occur at least every 3 years [69,70]. ^2^ No sample available for FCV and FHV. Bold: days since last vaccination <21 days and positive FCV and/or FHV results. FCV = feline calicivirus and FHV = feline herpesvirus.

**Table A2:** Clinician score for the general condition and severity of disease of a cat according to the Karnofsky’s score modified for cats by Hartmann and Kuffer [72].

Score	Description
0	Dead
10	Severely diseased
20	Major changes in the general condition
30	Medium changes in the general condition
40	Minor changes in the general condition
50	Completely normal general condition
